# Effect of Storage Temperature and Storage Time on the pH and Oxidation–Reduction Potential of Commercial Oral Moisturizers

**DOI:** 10.3390/dj13080344

**Published:** 2025-07-24

**Authors:** Mamoru Murakami, Sara Komabashiri, Kae Harada, Takaharu Shimizu, Masahiro Nishimura

**Affiliations:** 1 Removable Prosthodontics and Implant Dentistry, Advanced Dentistry Center, Kagoshima University Hospital, Kagoshima 890-8544, Japan; 2 Section of Dental Hygiene, Division of Clinical Technology, Kagoshima University Hospital, Kagoshima 890-8544, Japan; k9604974@dent.kagoshima-u.ac.jp; 3 Department of Prosthetic Dentistry, Medical and Dental Sciences, Graduate School of Biomedical Sciences, Nagasaki University, Nagasaki 852-8588, Japan; hkae@nagasaki-u.ac.jp; 4 Departments of Oral and Maxillofacial Prosthodontics, Field of Oral and Maxillofacial Rehabilitation, Advanced Therapeutic Course, Kagoshima University Graduate School of Medical and Dental Sciences, Kagoshima 890-8544, Japan; shimizu@dentshimizu.com; 5 Department of Regenerative Prosthodontics, Graduate School of Dentistry, Osaka University, Osaka 565-0871, Japan; nishimura.masahiro.dent@osaka-u.ac.jp

**Keywords:** oral care, pH, xerostomia

## Abstract

**Background/Objective**: The criteria for selecting and managing oral moisturizers have yet to be clearly defined. The purpose of this study was to examine the effects of storage temperature and storage time on the pH and oxidation–reduction potential (ORP) of oral moisturizers. **Methods**: The pH and ORP of 20 commercially available oral moisturizers stored at 37 °C, 25 °C, and 4 °C were measured immediately after opening (0M) and at 1 month (1M), 3 months (3M), and 6 months (6M) post-opening. The data were analyzed using Fisher’s exact test based on the critical pH of enamel and dentin, two-way repeated-measures analysis of variance (ANOVA), and Pearson’s correlation analysis. **Results**: At 0M, 25% of the products had pH values below the critical threshold for enamel, and 75% fell below that for dentin. The distribution of products significantly differed based on critical pH thresholds (*p* < 0.05). The two-way repeated-measures ANOVA showed that the pH was significantly affected by the storage time (*p* < 0.05), but not by the storage temperature or its interaction with time. By contrast, ORP was significantly affected by the storage temperature, storage time, and their interaction (*p* < 0.05). The Pearson’s correlation analysis revealed a significant negative correlation between pH and ORP at 4 °C (6M), 25 °C (1M, 3M, 6M), and 37 °C (all time points) (*p* < 0.05). **Conclusions**: Many oral moisturizers exhibit acidic pH values, indicating that products with a pH of 6.7 or higher should be selected. Additionally, to minimize degradation, oral moisturizers should be stored at 4 °C and used within 3 months of opening.

## 1. Introduction

In recent years, the number of patients with xerostomia has been increasing, with more than 30% of individuals aged ≥65 years affected. The number of potential patients is estimated to be as high as 30 million [1,2]. Xerostomia can be caused by a variety of factors, including aging, radiation therapy, and multidrug therapy [3,4].

In addition to experiencing dry mouth, individuals with xerostomia often suffer from worsening dental caries and periodontal disease, denture stomatitis, and an increased presence and detection rate of Candida, all of which contribute to a decline in quality of life [2,3,4,5,6]. Also, xerostomia has been reported to cause changes in the oral microflora [5,6,7,8,9,10]. Therefore, appropriate management is essential for dry mouth patients to reduce the risk of systemic infection [5].

For xerostomia caused by radiation therapy or Sjögren’s syndrome, medications such as pilocarpine hydrochloride and cevimeline hydrochloride are prescribed to stimulate saliva secretion. However, these drugs can cause side effects, including excessive sweating and gastrointestinal issues [11,12]. As a result, many patients with xerostomia use gel or liquid oral moisturizers as a form of symptomatic treatment [13,14,15,16,17,18,19].

Nevertheless, the criteria for selecting oral moisturizers have yet to be clearly defined. Given that patients with xerostomia experience reduced salivary secretion and acidified saliva [1,20,21,22], the pH of oral moisturizers may be a key factor in improving the oral environment affected by the condition. However, the pH of commercially available products is not disclosed, and only a few studies have examined the relationship between the pH of these products and erosion [23]. Therefore, a comprehensive investigation into the pH of oral moisturizers is essential.

On the other hand, while manufacturers’ instructions recommend storing oral moisturizers away from high temperatures and direct sunlight, they do not specify exact storage temperatures or durations. Previous studies on the viscosity and antifungal properties of oral moisturizers have shown that these properties can change depending on the storage temperature and time [13,14,24]. Similarly, the quality of food and beverages declines due to oxidation caused by heat-related deterioration or prolonged storage. As a result, quality assessments have been conducted using changes in pH and oxidation–reduction potential (ORP) [25,26,27]. While pH serves as an index for changes in hydrogen ion concentration, ORP reflects the oxidative or reductive state of the solution. ORP is influenced by physical factors such as temperature and the viscosity of the material measured, and a more positive ORP value indicates a stronger oxidizing power [28].

In this study, we focused on pH and ORP, which are considered indicators of changes in the properties of oral moisturizers, and examined their potential use in selecting and managing these products. We hypothesized that the storage temperature and duration was not affected on their pH and ORP. The aim was to investigate the pH of commercially available oral moisturizers and to assess the effects of the storage temperature and duration on their pH and ORP.

## 2. Materials and Methods

### 2.1. Samples, Storage Temperature, and Storage Time

Twenty commercially available oral moisturizers were used in this study, including nine liquid or spray types and eleven gel types. Details of the oral moisturizers are provided in Table 1 (liquid or spray) and Table 2 (gel). We used the same LOT number for all products. Unopened samples were stored in an incubator (Direct Heat CO_2_ Incubator 310; Thermo Fisher Scientific KK, Tokyo, Japan) at 37 °C and 25 °C and in a refrigerator (SJ-PW35Y-W; SHARP, Osaka, Japan) at 4 °C, starting 1 week prior to the experiment. The pH and ORP of each moisturizer were measured at four time points: immediately after opening (0M), 1 month after opening (1M), 3 months after opening (3M), and 6 months after opening (6M).

### 2.2. pH and ORP Measurement

pH and ORP were measured using a glass electrode-type hydrogen ion concentration meter (LAQUA act D72; Horiba, Kyoto, Japan). For pH measurements, a GRT composite electrode with a silver/silver chloride internal electrode (9681S-10D; Horiba) was used. For ORP measurements, a composite electrode with a platinum internal electrode (9300-10D; Horiba) was used. The measurement range, display resolution, and measurement accuracy for pH were 0.00 to 14.00 pH, 0.01 pH, and ±0.01 pH, respectively. For ORP, these were −2000 to 2000 mV, 1 mV, and ±1 mV, respectively.

The pH electrode was calibrated using three standard solutions (pH 4.01, pH 7.00, and pH 10.01) before measurements. The ORP electrode was calibrated using standard solutions prepared by adding ORP standard powders—monopotassium phthalate and quinhydrone (160-22; Horiba), as well as potassium dihydrogen phosphate, disodium hydrogen phosphate, and quinhydrone (160-51; Horiba)—to 250 mL of ion-exchanged water.

A 4 mL sample of each product was transferred into 8 mL sterile tubes (60.542 SX; Zalstat, Tokyo, Japan) stored at each designated temperature. To prevent temperature fluctuations during measurement, the tubes were placed in a small incubator (BBCW-1; As One, Osaka, Japan) set to match the storage temperature of the samples. According to the previous report [29], each sample was measured five times (*n* = 5), and the average value was calculated.

### 2.3. Statistical Analysis

A two-way analysis of variance (ANOVA) was conducted to examine the effects of the oral moisturizer type (liquid or gel) and storage temperature (4 °C, 25 °C, and 37 °C) on pH at 0M. The resulting pH values were further analyzed using cross-tabulation and Fisher’s exact test based on the critical pH thresholds for enamel and dentin. pH values were classified as acidic if <6.5 and neutral if ≥6.5 and <7.5 [29]. The critical pH was set at 5.5 for enamel and 6.7 for dentin and cementum [23].

To assess the effects of storage temperature and storage time on pH and ORP, a two-way repeated-measures ANOVA was performed, with the pH and ORP as dependent variables, storage temperature as the between-subject factor, and storage time as the within-subject factor. Tukey’s multiple comparisons test was used for the post hoc analysis.

The relationship between pH and ORP at each storage temperature and time point was evaluated using Pearson’s correlation analysis. All statistical analyses were performed using IBM SPSS Statistics 29 (Japan IBM, Tokyo, Japan), with the significance level set at *p* < 0.05.

## 3. Results

### 3.1. pH of Oral Moisturizers

Table 3 presents the pH values of oral moisturizers at each storage temperature at 0M (upper row) along with the results of the two-way ANOVA (lower row). At 4 °C, the maximum and minimum pH values were 7.80 and 2.87, respectively, and 65% of the products were classified as acidic. The two-way ANOVA performed at 0M revealed no significant differences in pH based on the type of oral moisturizer (gel or liquid), storage temperature, or the interaction between these two factors.

**Figure 1:**
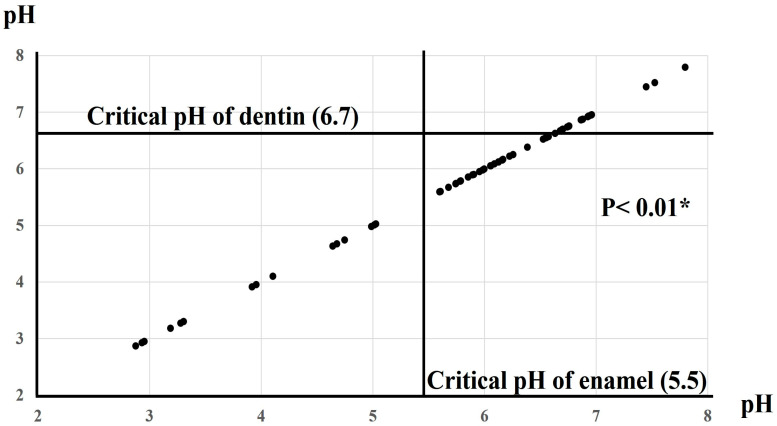
Relationship between the pH of oral moisturizers and the critical pH thresholds for enamel and dentin. Number (%). * *p* < 0.01: Fisher’s exact test.

Figure 1 illustrates the results of the cross-tabulation analysis of pH at 0M based on the critical thresholds for enamel and dentin. At this time point, 25% of the products had pH values below the critical level for enamel (5.5), while 75% fell below the critical level for dentin (6.7). The five products with the lowest pH values were identified as e (2.87), G (3.19), j (3.92), E (4.64), and a (4.99). By contrast, among the 15 products (25.0%) with pH levels above the critical thresholds for both enamel and dentin, the five highest were c (7.80), H (6.96), I (6.93), i (6.88), and C (6.76). Fisher’s exact test showed a statistically significant difference in the number of oral moisturizers falling above or below the critical pH levels for enamel and dentin (*p* < 0.05).

### 3.2. Effects of Storage Temperature and Time on pH and ORP

Table 4 presents the pH of oral moisturizers at each storage temperature and time point (upper row), the results of the two-way repeated-measures ANOVA (middle row), and Tukey’s multiple comparisons (lower row). The maximum pH recorded was 5.83 (at 4 °C, 0M), while the minimum was 5.49 (at 37 °C, 6M). Overall, pH values tended to decrease over time at all storage temperatures. The two-way repeated-measures ANOVA revealed a significant effect of storage time on pH, but no significant effect of the storage temperature or interaction between temperature and time. Tukey’s multiple comparisons indicated a significant drop in pH between 0M and 1M, 3M, and 6M, as well as between 1M and both 3M and 6M. However, no significant difference was observed between 3M and 6M.

Table 5 shows the ORP values of oral moisturizers under the same conditions, along with the ANOVA results and Tukey’s comparisons. The highest ORP value was 255.31 (at 4 °C, 0M), and the lowest was 171.04 (at 37 °C, 3M). ORP tended to decline over time at 4 °C. At 25 °C and 37 °C, ORP initially decreased until 3M, and then increased at 6M, displaying a different temporal pattern compared to pH.

The two-way repeated-measures ANOVA indicated significant effects of the storage temperature, storage time, and their interaction on ORP. Multiple comparisons showed that, when comparing ORP across temperatures at the same time point, ORP at 4 °C was significantly higher than at 37 °C at both 0M and 6M and significantly higher than at both 25 °C and 37 °C at 1M and 3M. When comparing ORP over time at a fixed temperature, no significant change was observed at 4 °C. By contrast, at both 25 °C and 37 °C, ORP was significantly lower at 3M than at 0M.

**Figure 2:**
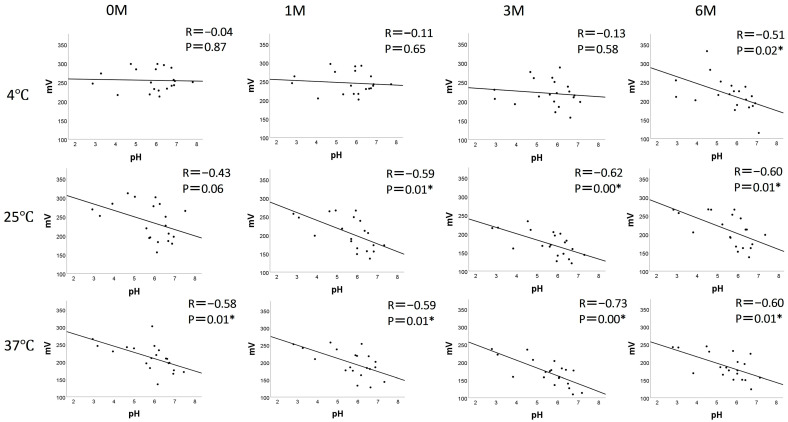
Correlation analysis between pH and ORP at each storage temperature and time point. * *p* < 0.05.

Figure 2 presents the results of the correlation analysis between pH and ORP across different storage temperatures and time points. A significant negative correlation between pH and ORP was found at 4 °C at 6M; at 25 °C at 1M, 3M, and 6M; and at 37 °C across all time points. These findings suggest that the higher the storage temperature, the stronger the negative correlation between pH and ORP, indicating that products with a lower pH are more prone to oxidation.

## 4. Discussion

A survey of dentists, dental hygienists, and nurses revealed that awareness of oral moisturizers and the ability to judge appropriate usage scenarios vary depending on the professional role and years of clinical experience [19]. Oral moisturizers are generally regarded as self-care products that patients purchase independently from supermarkets, drugstores, or online retailers. When selected and managed appropriately under the guidance of healthcare professionals, these products can be used effectively [19].

Oral moisturizers should have a lubricating effect, especially hydration lubrication. Proteins, glycoproteins, biopolymers, synthetic polymers, lipids, and phospholipids play a primary role in oral lubrication [30,31]. These are contained as active ingredients in various oral moisturizers [13,15,32]. For xerostomia patients who are able to take care of themselves, the dry feeling can be easily eliminated by using liquid-type products. On the other hand, using a gel moisturizer reduces the aspiration risk in older patients requiring nursing care or hospitalization [33]. However, clear criteria for selecting oral moisturizers along with standardized management practices—such as recommended storage temperatures and acceptable durations of use after opening—have yet to be established. To address this gap, the present study conducted a comprehensive analysis of the pH of commercially available oral moisturizers and investigated how the storage temperature and time after opening affect product quality. We used pH and ORP as indicators because they are considered reflective of changes in the properties of these products. It is the first study to systematically analyze the interaction between storage time (up to 6 months) and three temperatures. Also, we established a clear correlation between pH/ORP and storage-related degradation in a large sample.

### 4.1. Study Design and Measurement Methods

Although manufacturers generally advise storing oral moisturizers away from high temperatures and direct sunlight, they do not specify exact storage temperatures. In this study, we set the storage conditions to 4 °C (refrigeration), 25 °C (room temperature), and 37 °C (body temperature) to simulate a range of realistic storage environments. According to the Japanese Industrial Standards, room temperature is defined as 35 °C or lower. In this study, 37 °C, which corresponds to typical body temperature, was set as a comparatively higher temperature condition. While previous research has examined the effect of 37 °C storage on the residual weight of oral moisturizers, the impact of lower temperatures, such as 4 °C, has not been clearly elucidated [14]. To ensure temperature consistency across products with varying package thickness and size, all samples were stored at their respective temperatures for 1 week prior to measurement. The results of the two-way ANOVA at 0M suggested that the pH of oral moisturizers immediately after opening is not influenced by product type or storage temperature when pre-heated or stabilized for 1 week.

Regarding storage duration, some products include instructions to use the product immediately after opening. Among the 20 products tested in this study, 13 lacked labeling of either an expiration or manufacturing date. This may be due to Japanese regulations, which do not require such labeling for products that maintain stable quality for more than 3 years after manufacture. The remaining seven products did provide expiration or manufacturing dates. As a result, it is possible that the effect of storage temperature and time on pH and ORP may vary depending on whether the product includes an expiration date.

To measure pH, three primary methods are commonly used: the indicator method, the metal electrode method, and the glass electrode method [34]. This study employed the glass electrode method, which determines the pH based on the potential difference between a glass electrode and a reference electrode. This method is widely adopted because of its strong reproducibility and minimal susceptibility to interference from oxidizing or reducing agents [34]. The electrode used in this study was suitable for measuring highly viscous and non-aqueous samples, making it effective for assessing the pH of a broad range of oral moisturizers, including both liquid and gel types [34].

### 4.2. pH of Oral Moisturizers

A previous study examining the pH of seven commercially available oral moisturizers reported a minimum value of 3.03, a maximum of 9.09, and an average of 6.4 [23]. Another study evaluating 12 antifungal oral moisturizers found pH values ranging from 3.46 to 7.29, with an average of 6.26 [35]. In comparison, the 20 oral moisturizers assessed in this study showed a pH range from 2.87 to 7.80, which aligns with the ranges reported in prior research, although the average value in this study was notably lower at 5.83. This lower average and the finding that 65% of products had acidic pH values are likely due to the inclusion of food additives such as citric acid or trisodium citrate, which act as pH adjusters or microbial inhibitors, as well as acidic preservatives like sorbic acid and benzoic acid (see Table 1 and Table 2).

The acidic pH of many oral moisturizers is problematic for improving the oral environment in patients with xerostomia. Previous studies have explored the association between the low pH of oral moisturizers or denture adhesives and acid erosion [23,35]. Ideally, saliva substitutes should have a prolonged effect in the oral cavity, but patients with xerostomia often have a diminished salivary buffering capacity. This makes them more susceptible to tooth demineralization when using low-pH products [3,4]. In this study, the products identified as having pH values above the critical threshold for dentin were c, H, I, i, and C. For dentate patients with xerostomia—particularly those experiencing gingival recession—these products may be preferable to reduce the risk of root caries [1].

### 4.3. Effects of Storage Temperature and Time on pH and ORP

Previous studies have shown that the storage temperature and duration of oral moisturizers influence their viscosity and antifungal properties [13,14,24]. Similarly, the quality of drinking water and food products is known to deteriorate due to oxidation caused by thermal degradation and aging, with changes in pH and ORP commonly used to evaluate such quality shifts [25,26,27].

Because ORP evaluates the redox potential of any substance containing hydrogen ions, it is expressed as a function of the pH using the Nernst electrode potential equation [36]. This means that the same ORP value may represent different chemical states depending on the pH; thus, this study analyzed both the pH and ORP in parallel.

One study measuring the pH and ORP of pure and mineral water reported that, for pure water, the pH remained stable at 6.1 from 18 °C to 22 °C, but ORP increased markedly from 62 mV to 150 mV. Similarly, for mineral water, the pH remained at 8.3, but ORP rose from −164 mV to 62 mV as the temperature increased from 18 °C to 22 °C [28]. These findings align with our results, where the pH remained relatively unaffected by the storage temperature, while ORP showed a clear temperature-dependent variation. In particular, ORP values increased between 3M and 6M at 25 °C and 37 °C, likely due to the oxidation of product components driven by thermal degradation or aging over time.

The present study demonstrated that the pH and ORP in oral moisturizers are influenced differently by the storage temperature and time. These results suggest that both factors should be considered together when determining appropriate storage conditions. The correlation analysis further showed that, at 25 °C and 37 °C, products with a lower pH exhibited a higher tendency toward oxidation early in the storage period. This finding implies that, to minimize quality degradation, oral moisturizers should ideally be stored at 4 °C and used within three months of opening.

### 4.4. Limitations of the Study and Future Prospects

The pH indicates the strength of acidity in an aqueous solution resulting from the dissociation of acids in water, whereas the overall concentration of acid in the solution is referred to as acidity. Studies investigating the pH and neutralization titration of soft drinks have shown that these beverages often have low pH values—ranging from 2.81 to 3.45—and are associated with an increased risk of acid erosion. Notably, fruit juices and fruit-based carbonated drinks with high titratable acidity have been found to resist the buffering action of saliva, leading to a prolonged decrease in oral pH [36]. This suggests that even oral moisturizers with similar pH values may differ significantly in actual acidity. Because resistance to salivary buffering varies with the strength of acidity, it may be necessary in future research to evaluate the acid dissociation constant (pKa) of oral moisturizers. Techniques such as neutralization titration could help provide a more comprehensive understanding of the acidic potential and its implications for oral health.

In the present study, we did not quantitatively analyze the specific contribution of each additive to the acidity. Further studies are needed to determine a more detailed breakdown of how individual excipients affect the pH of these products. Also, we did not use clinical simulation at the natural saliva level in the present study. In the future, we need to use a much more clinically relevant approach in methodology. Alternatively, the application of natural substances such as postbiotics and the re-mineralizing effects of hydroxyapatite may provide benefits [37,38].

## 5. Conclusions

In light of the limitations of this in vitro study, it can be concluded that:

1. Because many oral moisturizers are acidic, products with a pH of 6.7 or higher should be prioritized.
2. To maintain quality and minimize degradation, oral moisturizers should be stored at 4 °C and used within 3 months after opening.

## Figures and Tables

**Table 1:** Details of the liquid- and spray-type oral moisturizers used in this study.

Materials	Code	Manufacturer (City, Country)	Main/Active Ingredients
Stoppers for	A	Sun Dental Co., Ltd. (Osaka, Japan)	Water, Glycerin, Xylitol, Lysozyme, Lactoferrin
Oral Wet Spray	B	Yoshida Co., Ltd. (Tokyo, Japan)	Water, Xylitol, Sodium benzoate, Hyaluronate sodium
ConCool Mouth Rinse	C	Weltec Co., Ltd. (Osaka, Japan)	Water, PG, Sorbitol, Xylitol, Lactoferrin, Whey protein
Pepti Sal Mouth Wash	D	T&K Co., Ltd. (Tokyo, Japan)	Water, PG, Xylitol, Polyglycitol, Nisin, Lactoferrin
Wet Keeping Mist	E	Oral Care. Inc. (Tokyo, Japan)	Glycerin, Betaine, Xylitol, Methylparaben, Sodium citrate
Biotene Mouth Wash	F	GSK Co., Ltd. (Tokyo, Japan)	Water, Glycerin, Xylitol, Sorbitol, Propylene glycol
Clean & Moisture Spray	G	Trife Inc. (Yokohama, Japan)	Water, Glycerin, Lactococcus culture extract, Ume extract
SMILE HONEY Gel Spray	H	Nippon Zettoc Co., Ltd. (Tokyo, Japan)	Water, Glycerin, Propanediol, Hyaluronate sodium, Xylitol
Sunstar Gel Spray	I	SUNSTAR Co., Ltd. (Osaka, Japan)	Water, Glycerin, Glycosyl trehalose, BG

**Table 2:** Details of the gel-type moisturizers used in this study.

Materials	Code	Manufacturer (City, Country)	Main/Active Ingredients
Wet aid	a	Dent care. Inc. (Osaka, Japan)	Water, Maltitol, Trehalose, BG, Hyaluronate sodium
Oral Moisturizer Ai Gel	b	Ryoka Dental Inc. (Mie, Japan)	Water, Glycerin, Carboxymethyl cellulose, Xylitol
Oral Aquagel	c	GC Co., Ltd. (Tokyo, Japan)	Diglycerine, Water, Carboxymethyl cellulose
Denture Gel	d	Kamemizu Chem. Ind. (Osaka, Japan)	Maltitol, Water, Glycerin, Propylene glycol
SMILE HONEY Gel	e	Nippon Zettoc Co., Ltd. (Tokyo, Japan)	Honey, Xylitol, Sucralose, Lemon juice, Tea extract
ConCool Mouth Gel	f	Weltec Co., Ltd. (Osaka, Japan)	Water, Maltitol, Sorbitol, Glycerin, Xylitol, Whey protein
Oral Balance Gel	g	GSK Co., Ltd. (Tokyo, Japan)	Glycerin, Water, Sorbitol, Xylitol, Hydroxyethyl cellulose
Terumo Oral Gel	h	Nippon Zettoc Co., Ltd. (Tokyo, Japan)	Water, Sorbitol, Glycerin, Xylitol, Dried egg yolk, Maltitol
Butler Moisturizing Gel	i	SUNSTAR Co., Ltd. (Osaka, Japan)	Water, Glycerin, Sorbitol, Hydrogenated starch hydrolysate
Clean & Moisture Gel	j	Trife Inc. (Yokohama, Japan)	Glycerin, Water, Lactococcus culture extract, Ume extract
Care Heart Gel	k	Tamagawa Eizai Co., Ltd. (Tokyo, Japan)	Water, Glycerin, Xylitol, Hydroxyethyl cellulose

**Table 3:** pH values of oral moisturizers at each storage temperature at 0M and results of two-way ANOVA (* mean (SD), the significance level set at *p* < 0.05).

	4 °C	25 °C		37 °C	
Liquid *	5.94 (1.22)	5.89 (1.20)		5.89 (1.25)	
Gel *	5.74 (1.34)	5.67 (1.30)		5.66 (1.28)	
Liquid and Gel *	5.83 (1.26)	5.77 (1.23)		5.76 (1.24)	
Minimum value	2.87	2.93		2.95	
Maximum value	7.80	7.52		7.45	
Acidic/Neutral/Alkaline (Acid%)	13/6/1 (65%)	13/6/1 (65%)		13/7/0 (65%)	
Source	Sum of Squares	Degrees of Freedom	Mean Square	f-Value	*p*-Value
Type of moisturizers (A)	0.68	1	0.68	0.42	0.52
Storage temperature (B)	0.05	2	0.02	0.01	0.99
(A) × (B)	0.00	2	0.00	0.00	0.99
Error	87.16	54	1.61		
Total	2096.78	60			

**Table 4:** pH values of oral moisturizers at each storage temperature and time point (upper row), results of two-way repeated-measures ANOVA (middle row), and results of Tukey’s multiple comparisons (lower row). Upper row, Mean (SD); Lower row, Tukey (* *p* < 0.05): 0M (5.7) > 1M (5.69) > 3M (5.58), 6M (5.54).

	0M	1M	3M	6M	
4 °C	5.83 (1.26)	5.73 (1.30)	5.60 (1.20)	5.58 (1.24)	
25 °C	5.77 (1.23)	5.65 (1.22)	5.58 (1.21)	5.56 (1.21)	
37 °C	5.76 (1.24)	5.69 (1.22)	5.56 (1.21)	5.49 (1.20)	
Source	Sum of Squares	Degrees of Freedom	Mean Square	f-Value	*p*-Value
Storage time (A)	2.17	2.21	0.98	67.99	0.00 *
Storage temperature (B)	0.15	2	0.08	0.01	0.99
(A) × (B)	0.07	4.42	0.02	1.03	0.40
Error	343.75	182.83	1.88		
Total	346.14	191.50			
	0M	1M	3M	6M	
0M	-	0.00 *	0.00 *	0.00 *	
1M	-	-	0.00 *	0.00 *	
3M	-	-	-	0.57	
6M	-	-	-	-	

**Table 5:** ORP values of oral moisturizers at each storage temperature and time point (upper row), results of two-way repeated-measures ANOVA (middle row), and results of Tukey’s multiple comparisons (lower row). * *p* < 0.05. Upper row, Mean (SD), * *p* < 0.05. Vertically, different symbols (#, $) denote significant differences between storage temperature. Horizontally, different alphabet letters (A,B) denote significant differences between storage time (Tukey: *p* < 0.05).

	0M	1M	3M	6M	
4 °C	255.31 (28.50)	245.69 (29.42)	221.03 (34.11)	217.66 (44.41)	
25 °C	237.01 (48.44)	205.10 (43.58)	173.44 (33.07)	210.67 (43.27)	
37 °C	213.54 (38.69)	198.20 (39.62)	171.04 (36.80)	188.12 (36.41)	
Source	Sum of Squares	Degrees of Freedom	Mean Square	f-Value	*p*-Value
Storage time (A)	69,248.52	2.38	29,043.16	66.07	0.00 *
Storage temperature (B)	74,043.01	2	37,021.51	7.59	0.00 *
(A) × (B)	11,160.08	4.769	2340.30	5.32	0.00 *
Error	337,664.50	192.91	1750.40		
Total	492,116.12	202.06			
	0M	1M	3M	6M	
4 °C	# A	# A	# A	# A	
25 °C	# A	$ B	$ B	# B	
37 °C	$ A	$ B	$ B	$ B	

## Data Availability

The original contributions presented in this study are included in the article.
